# Regulation of cAMP and GSK3 signaling pathways contributes to the neuronal conversion of glioma

**DOI:** 10.1371/journal.pone.0178881

**Published:** 2017-11-21

**Authors:** Jinsoo Oh, Yongbo Kim, Lihua Che, Jeong Beom Kim, Gyeong Eon Chang, Eunji Cheong, Seok-Gu Kang, Yoon Ha

**Affiliations:** 1 Department of Neurosurgery, Spine & Spinal Cord Institute, College of Medicine, Yonsei University, Seoul, South Korea; 2 Brain Korea 21 PLUS Project for Medical Science, Yonsei University College of Medicine, Seoul, South Korea; 3 Hans Schöler Stem Cell Research Center (HSSCRC), School of Life Sciences, Ulsan National Institute of Science and Technology (UNIST), Ulsan, South Korea; 4 Max Planck Partner Group-Molecular Biomedicine Laboratory (MPPG-MBL), UNIST, Ulsan, South Korea; 5 Department of Biotechnology, College of Life Science and Biotechnology, Yonsei University, Seoul, South Korea; Swedish Neuroscience Institute, UNITED STATES

## Abstract

Glioma is the most malignant type of primary central nervous system tumors, and has an extremely poor prognosis. One potential therapeutic approach is to induce the terminal differentiation of glioma through the forced expression of pro-neural factors. Our goal is to show the proof of concept of the neuronal conversion of C6 glioma through the combined action of small molecules. We investigated the various changes in gene expression, cell-specific marker expression, signaling pathways, physiological characteristics, and morphology in glioma after combination treatment with two small molecules (CHIR99021, a glycogen synthase kinase 3 [GSK3] inhibitor and forskolin, a cyclic adenosine monophosphate [cAMP] activator). Here, we show that the combined action of CHIR99021 and forskolin converted malignant glioma into fully differentiated neurons with no malignant characteristics; inhibited the proliferation of malignant glioma; and significantly down-regulated gene ontology and gene expression profiles related to cell division, gliogenesis, and angiogenesis in small molecule–induced neurons. *In vivo*, the combined action of CHIR99021 and forskolin markedly delayed neurological deficits and significantly reduced the tumor volume. We suggest that reprogramming technology may be a potential treatment strategy replacing the therapeutic paradigm of traditional treatment of malignant glioma, and a combination molecule comprising a GSK3 inhibitor and a cAMP inducer could be the next generation of anticancer drugs.

## Introduction

It has been reported that mouse fibroblasts can be directly reprogrammed into neurons through the forced expression of three pro-neuronal transcription factors (Brn2, Ascl1, and Myt1l) [1]. Indeed, cell reprogramming techniques have progressed rapidly and are breaking new ground in the field of regenerative medicine [2–9]. Glioma is the most malignant type of primary tumors, and has an extremely poor prognosis [10,11]. The direct conversion from malignant to benign cells through the forced expression of pro-neural factors is a potent therapeutic strategy for removing tumor cells because terminally differentiated-cells will die from apoptosis [12,13].

However, limitations in gene transfection efficiency for multiple-gene reprogramming techniques have led researchers to investigate strategies that replace or decrease the number of necessary reprogramming factors [14]. To this end, it was recently reported that somatic cells can be converted into neurons through the forced expression of a single key factor, such as Ascl1 or Neurod1 [15,16]. Despite this progress, intrinsic limitations related to chromosomal gene insertion persist. Further, it was recently reported that mouse and human fibroblasts could be converted into neurons using a small molecule cocktail of 4–7 molecules *in vitro* [17,18].

We observed the development of neuron-like cells in a glioma culture following the addition of a small-molecule mixture containing the GSK3 inhibitor CHIR99021 and the cAMP activator forskolin, without forced-gene expression. Here, we investigated the various changes relating to both neural differentiation and growth suppression after neural conversion, as well as the signaling pathways involved in glioma–neural conversion by CHIR99021 and forskolin. Further, we investigated whether direct reprogramming technology based on the combination of small molecules can be applied to the treatment of malignant glioma. This study is the first to show that glioma can be converted to neurons using small molecules without neural transcription factors.

## Materials and methods

### Direct reprogramming of glioma to neurons using small molecules

Rat C6 glioma cells were cultured in DMEM (Invitrogen) containing 10% FBS (Hyclone) and 1% P/S (Invitrogen). C6 glioma cells have been tested previously [19]. Glioblastoma (GBM) was established from patients as approved by the institutional review board of Severance Hospital, Yonsei University College of Medicine [20]. GBM was cultured in DMEM/F12 (Corning) containing 2% B27, 1% P/S (Invitrogen), epidermal growth factor (EGF; 10 ng/ml) and fibroblast growth factor 2 (FGF2; 10 ng/ml). For neuronal induction, C6 glioma was plated at a density of 2 × 10^3^ cells/cm^2^ on a matrigel (BD Bioscience)-coated 12-mm glass coverslip. GBM were plated at density of 4 × 10^4^ cells/cm^2^ on a matrigel (BD Bioscience)-coated 12-mm glass coverslip. The following day, media were replaced with neural induction media consisting of DMEM/F12 (Invitrogen): neurobasal medium (Invitrogen) (1:1 mixture) containing 1% N2 (Invitrogen) and 2% B27 (Invitrogen) supplement, 1× Glutamax (Invitrogen), 0.05% bovine serum albumin (BSA), β-mercaptoethanol, 1% P/S (Invitrogen), brain-derived neurotrophic factor (BDNF; 10 ng/mL, Peprotech), and glial cell-derived neurotrophic factor (GDNF; 10 ng/mL, Peprotech). Two small molecules (CHIR99021 and forskolin) were added into the neural induction media. The medium was replaced every 2~3 days.

In some experiments, mouse embryonic fibroblasts (MEFs) were used instead of glioma. MEFs were seeded at a density of 1 × 10^4^ cells/cm^2^ on a matrigel-coated 12-mm glass coverslip. The following day, neural induction was performed, as described above.

### Cell proliferation and toxicity assay

C6 glioma and GBM were cultured in culture medium containing dimethyl sulfoxide (DMSO), CHIR99021 (2-20 μM), forskolin (10-100 μM), or a combination of CHIR99021 and forskolin. On the day of the experiment, the medium was replaced with culture medium containing 3-(4,5-dimethylthiazol-2-yl)-2,5-diphenyltetrazolium bromide (MTT) reagent (Sigma) at a final concentration of 0.5 mg/mL, and incubated for 3 hours. Violet crystal was dissolved in DMSO and added to the wells. Absorbance was measured using an enzyme-linked immunosorbent assay (ELISA) plate reader (VERSAmax) at 490 nm. To test the cell toxicity, the number of viable cells was counted by trypan blue staining after DMSO or small-molecule treatment.

### Immunocytochemistry

Cells were washed with phosphate-buffered saline (PBS) and then fixed in 4% paraformaldehyde (Merck) for 10 minutes. Cells were then washed with PBS three times for 3 minutes per wash, and subsequently incubated with a blocking solution (10% normal donkey serum, Jackson ImmunoResearch) for 1 hour at room temperature (RT). Cells were incubated with primary antibodies for 1 hour at RT, washed three times with PBS, incubated with secondary antibody for 30 minutes at RT, and finally washed three more times and mounted with a mounting solution (Vector Laboratories). Cells were analyzed using an Olympus BX51 fluorescence microscope (Olympus) and an LSM 700 confocal microscope (ZEISS). The primary antibodies used in this study were: Tuj1 (1:2000, Abcam), MAP2 (1:500, Abcam), Nestin (1:1000, Millipore), GFAP (1:1000, Abcam), NG2 (1:200, Millipore), Olig2 (1:200, Abcam), platelet-derived growth factor receptor (PDGFR; 1:500, Santa Cruz) and Ki67 (1:250, Abcam).

### Whole-cell patch clamp

A cover slip with cultured cells was transferred to a recording chamber (Warner Instruments) and placed under a microscope (Olympus). Artificial CSF, containing 124 mM NaCl, 3 mM KCl, 1.3 mM MgSO_4_, 1.25 mM NaH_2_PO_4_, 26 mM NaHCO_3_, 2.4 mM CaCl_2_-2H_2_O, and 10 mM glucose (Sigma-Aldrich) was continuously perfused over the cells and aerated with O_2_ 95%/CO_2_ 5% mixed gas at RT. A glass capillary pipette was directed towards the cell surface. Negative pressure was provided to elicit a gigaseal for whole-cell recording. The internal pipette solution contained 115 mM K-gluconate, 10 mM KCl, 10 mM HEPES, 10 mM EGTA, 5 mM Mg-ATP, and 0.5 mM Na^2+^-GTP (Sigma-Aldrich), with pH 7.3 and 280–285 mOsm. Sodium current was recorded in voltage clamp mode, and electrical stimulation was provided in the range −60 mV to +20 mV. Thereafter, current clamp mode was utilized to evaluate action potential generation. To verify that inward currents and spikes were mediated by voltage gated sodium channels, the bath was treated with tetrodotoxin (TTX; 0.5 μM, Sigma-Aldrich) prior to recording. Data acquisition was performed using Digitizer 1440A (Molecular Devices) and Clampex 10.3 (Molecular Devices). Analysis of data was conducted by using Clampfit 10.3 (Molecular Devices).

### DNA microarray analysis

Microarray analysis was performed according to the manufacturer’s instructions. After total RNA isolation, cDNA was synthesized using the GeneChip WT (Whole Transcript) Amplification kit. Labeled DNA target was hybridized to the Affymetrix GeneChip Array. Hybridized arrays were washed and stained on a GeneChip Fluidics Station 450 and scanned on a GCS3000 Scanner. Analysis was performed using Affymetrix® GeneChip Command Console® Software (AGCC). Microarray data have been deposited in a public database (https://www.ncbi.nlm.nih.gov/geo/, GSE98656).

### Intramedullary spinal cord tumor (IMSCT) model and small molecule administration

Adult male C57BL mice (6 weeks, Orient Bio) were used for the IMSCT model. Zoletil 50 (30 mg/kg, Virbac) and Rompun (10 mg/kg, Bayer) were used for anesthesia. To establish the IMSCT, laminectomy was performed at the 10^th^ thoracic level. For *in vivo* tracing, we used the red fluorescent protein (RFP)-expressing C6 glioma [19]. Total 1 × 10^5^ cells of RFP-expressing C6 glioma were injected at a rate of 1 μl/min. Two days after inoculation, animals were divided into two groups: Group 1, water; Group 2, CHIR99021 (12.5 mg/kg, intraperitoneally) and forskolin (10 mg/kg, orally) once a day. To test neurological function, we observed hind limb movements based on Basso Mouse Scale (BMS) score every day following the glioma transplant.

### *In vivo* imaging system (IVIS)

The IVIS Spectrum (Xenogen, Alameda, CA, USA) was used to measure a cell mass of RFP-expressing C6 glioma. Sixteen days after glioma transplantation, the spinal cord was dissected, and bioluminescence detection was conducted for 1 minute. Regions of interest (ROIs) were analyzed, and total quantification of bioluminescence was performed using Living Image^®^ (Xenogen) software.

### Western blot

On the day of the experiment, equal amounts of protein sample were loaded on an 8-10% sodium dodecyl sulfate (SDS) gel and separated using SDS polyacrylamide gel electrophoresis (SDS-PAGE). Then, the protein samples were transferred to a polyvinylidene difluoride (PVDF) membrane for 1–2 hours at 100 V. After a blocking step with PBS containing 3% BSA, the transferred membrane was incubated with primary antibody. The following day, the membrane was incubated with horseradish peroxidase (HRP)-conjugated secondary antibody for 1 hour, and then the detection procedure was performed. Primary antibodies were: stage-specific embryonic antigen 1 (SSEA-1; 1:1000, Invitrogen), actin (1:10000, Abcam), and C-Myc (1:500, Santacruz).

### Statistical analysis

Student’s t-test was performed to assess differences between two groups. Two-way analysis of variance (ANOVA) was performed to assess differences among three groups or more. Data are presented as the mean ± standard error of the mean (SEM) and *p*-values less than 0.05 were considered to be statistically significant.

### Study approval

All experiments were performed according to international guidelines on the ethical use of animals, and the number of animals used was minimized. All protocols were approved by the Yonsei University Health System Institutional Animal Care and Use Committee (YUHS-IACUC). The patient provided written consent for their sample to be used in this study.

## Results

### The flexible neuronal conversion of malignant C6 glioma through the action of small molecules

**Fig 1:**
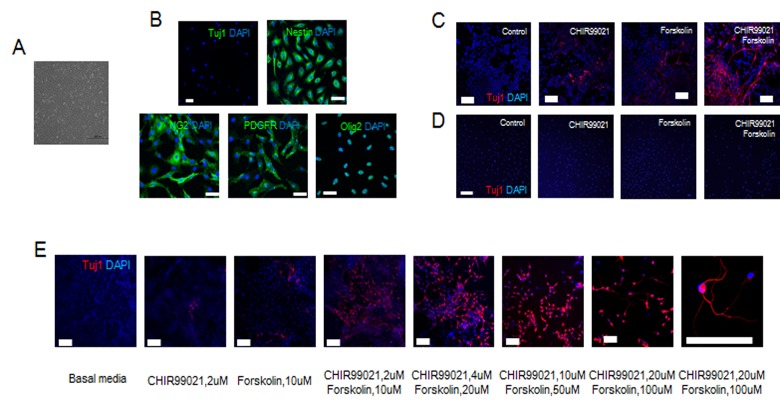
The flexible neuronal conversion of malignant C6 glioma through the action of small molecules. (A and B) Representative images of C6 glioma before neural induction. (C) Representative fluorescence image of Tuj1-positive cells 7 days after neural induction of glioma with CHIR99021 (2 μM) and forskolin (10 μM). (D) Representative image of Tuj1/DAPI staining at 7 days after treatment of mouse embryonic fibroblasts with a combination of CHIR99021 (2 μM) and forskolin (10 μM). (E) Representative image of Tuj1-positive cells 7 days after treatment of glioma with a variety of concentrations of CHIR99021 and forskolin. Scale bars represent 500 μm (A), 50 μm (B), 100 μm (C), 100 μm (D), and 100 μm (E).

In this study, we determined that malignant glioma cells can be converted into fully differentiated neurons through the combined action of two small molecules (CHIR99021, a GSK3 inhibitor, and forskolin, a cAMP activator) in the absence of neural transcription factors. The typical morphology of a C6 glioma is shown in Fig 1A. The majority of C6 glioma cells strongly expressed nestin and oligodendrocyte precursor cell markers such as PDGFR-α, NG-2, and Olig2, but not the neuron-specific marker Tuj1 (Fig 1B). Seven days after treatment with CHIR99021 or forskolin, Tuj1-positive cells were noted on C6 glioma, although the observed conversion efficiency was low (Fig 1C). In contrast, we confirmed that fibroblasts could not be converted into Tuj1-positive neurons by small molecule combination or single treatment (Fig 1D). Combined CHIR99021 and forskolin showed high efficiency conversion from glioma to Tuj1-positive cells (Fig 1C). We thus selected the combination of CHIR99021 and forskolin for further investigation. Seven days after the treatment with 20 μM CHIR99021 and 100 μM forskolin, we observed a marked increase in the conversion efficiency of malignant C6 glioma to Tuj1-positive cells (Fig 1E).

### Direct reprogramming of C6 glioma to neurons using two small molecules

**Fig 2:**
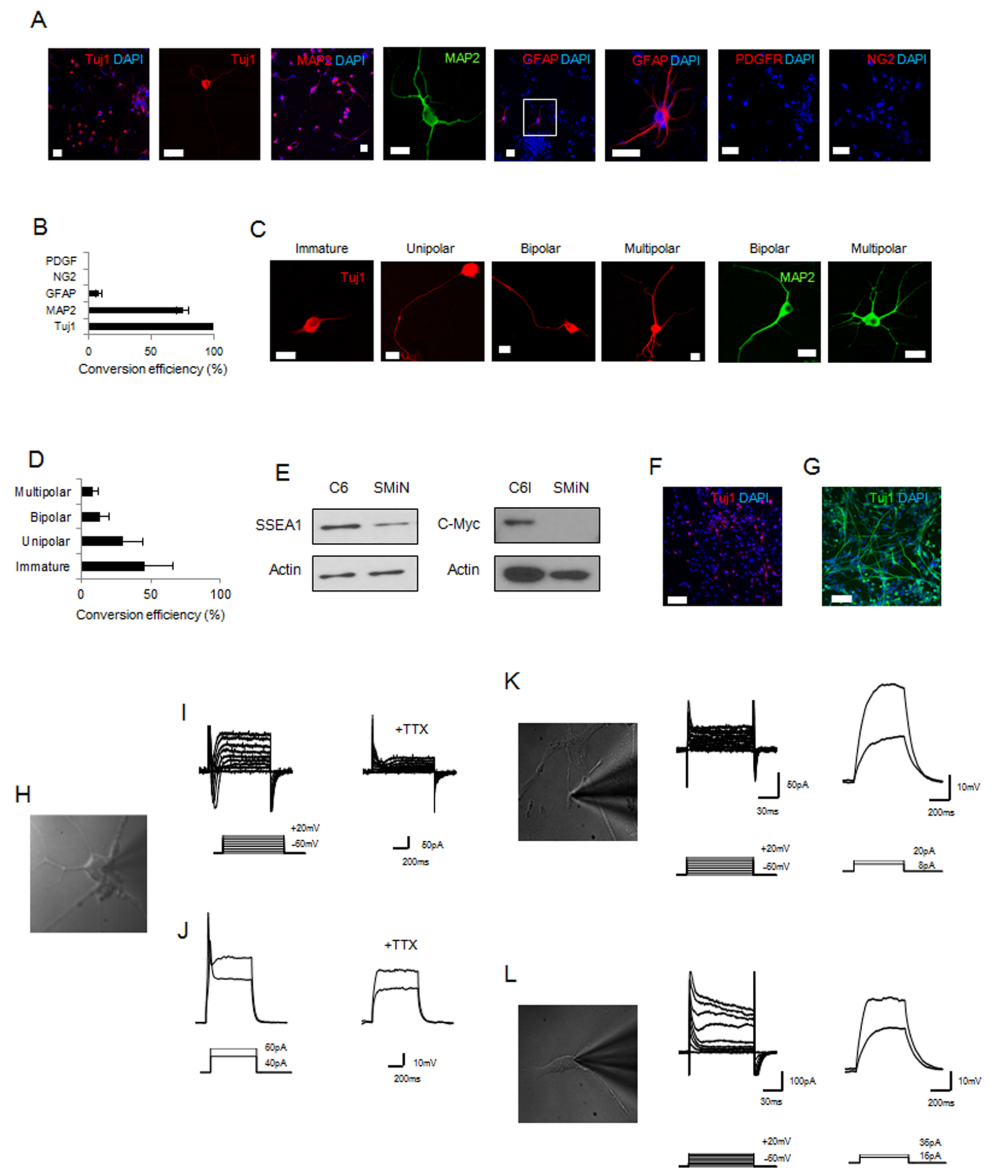
Direct reprogramming of C6 glioma to neurons using two small molecules. (A) Representative fluorescence images of small molecule-induced neurons (SMiNs) 35 days after neural induction with CHIR99021 (20 μM) and forskolin (100 μM). (B) Quantitative result of Tuj1, MAP2, GFAP, PDGFR, and NG2 staining in SMiNs 35 days after neural induction. (C) Representative image of Tuj1- or MAP2-positive SMiNs 35 days after neural induction with CHIR99021 (20 μM) and forskolin (100 μM). (D) Quantitative result of morphological complexity of Tuj1-positive SMiNs 35 days after neural induction. (E) Representative band image of SSEA1 (a cancer stem cell marker) and C-Myc (an oncogene marker) 35 days after neural induction with CHIR99021 (20 μM) and forskolin (100 μM). (F) Representative fluorescence images of SMiNs 7 days after neural induction with lithium carbonate (3 mM) and forskolin (100 μM). (G) Representative fluorescence images of SMiNs 7 days after neural induction with CHIR99021 (2 μM) and dbcAMP (0.5 mM). (H) Representative images of patch-clamp in SMiNs. (I) Representative traces of sodium current of SMiN 84 days after neural induction before (left) and after (right) 0.5μM TTX bath application. (J) Representative traces of action potential spike of SMiN 84 days after neural induction before (left) and after (right) 0.5μM TTX bath application. (K) Representative traces of sodium current (left) and action potential spike (right) of undifferentiated C6 glioma in proliferation media. (L) Representative traces of sodium current (left) and action potential spike (right) of undifferentiated C6 glioma in neural differentiation media without small molecules. Data are presented as the mean ± S.E.M. Scale bars represent 50 μm (A), 20 μm (C), 100 μm (F), and 100 μm (G).

Thirty-five days after neural induction with CHIR99021 and forskolin, most of the glioma was converted into Tuj1-positive (~90%) or MAP2-positive (~80%) cells showing morphological diversity (Fig 2A–2D). Some cells (~10%) had differentiated into GFAP-positive cells. However, cells expressing glioma markers PDGFR or NG2 were rarely observed 35 days after small-molecule treatment (Fig 2A and 2B). We compared the protein expression pattern of oncogene and cancer stem cell markers in C6 glioma and small molecule-induced neurons (SMiNs). The expression level of SSEA1 (a cancer stem cell marker) and C-Myc (an oncogene marker) was lower in SMiN compared with C6 glioma (Fig 2E). This result indicates that the combination treatment of CHIR99021 and forskolin changed the tumor characteristics. In addition, we investigated whether another GSK3 inhibitor (lithium carbonate) and cAMP activator (dbcAMP) induced neural conversion in malignant glioma. We found that the combination of lithium carbonate/forskolin or CHIR99021/dbcAMP was able to induce the neural conversion of glioma (Fig 2F and 2G). These results suggest that a malignant glioma can be converted into post-mitotic neurons through the application of a GSK3 inhibitor and a cAMP activator, especially CHIR99021 and forskolin, in the absence of neural transcription factors or forced gene expression. To examine whether SMiNs differentiated from malignant glioma demonstrated neuronal functionality, their electrophysiological properties were examined by whole-cell patch clamp recording (Fig 2H). In voltage clamp mode, Na^+^ currents were observed in SMiNs (Fig 2I, left). Action potential spikes appeared only in cells that produced Na^+^ currents (Fig 2J, left). To confirm that the channels producing currents and spikes were Na^+^ channels, the Na^+^ channel antagonist tetrodotoxin (TTX) was applied for 5–10 minutes. TTX treatment completely blocked Na^+^ currents (Fig 2I, right) and action potential spikes (Fig 2J, right). We did not see any signal in undifferentiated glioma cultured in proliferation (Fig 2K) and neural differentiation (Fig 2L) media. Thus, electrophysiology results showed that the characteristic of C6 glioma changed to that of functional neurons after combined treatment with the two small molecules.

### Growth suppression effect of a combination of CHIR99021 and forskolin *in vitro*

**Fig 3:**
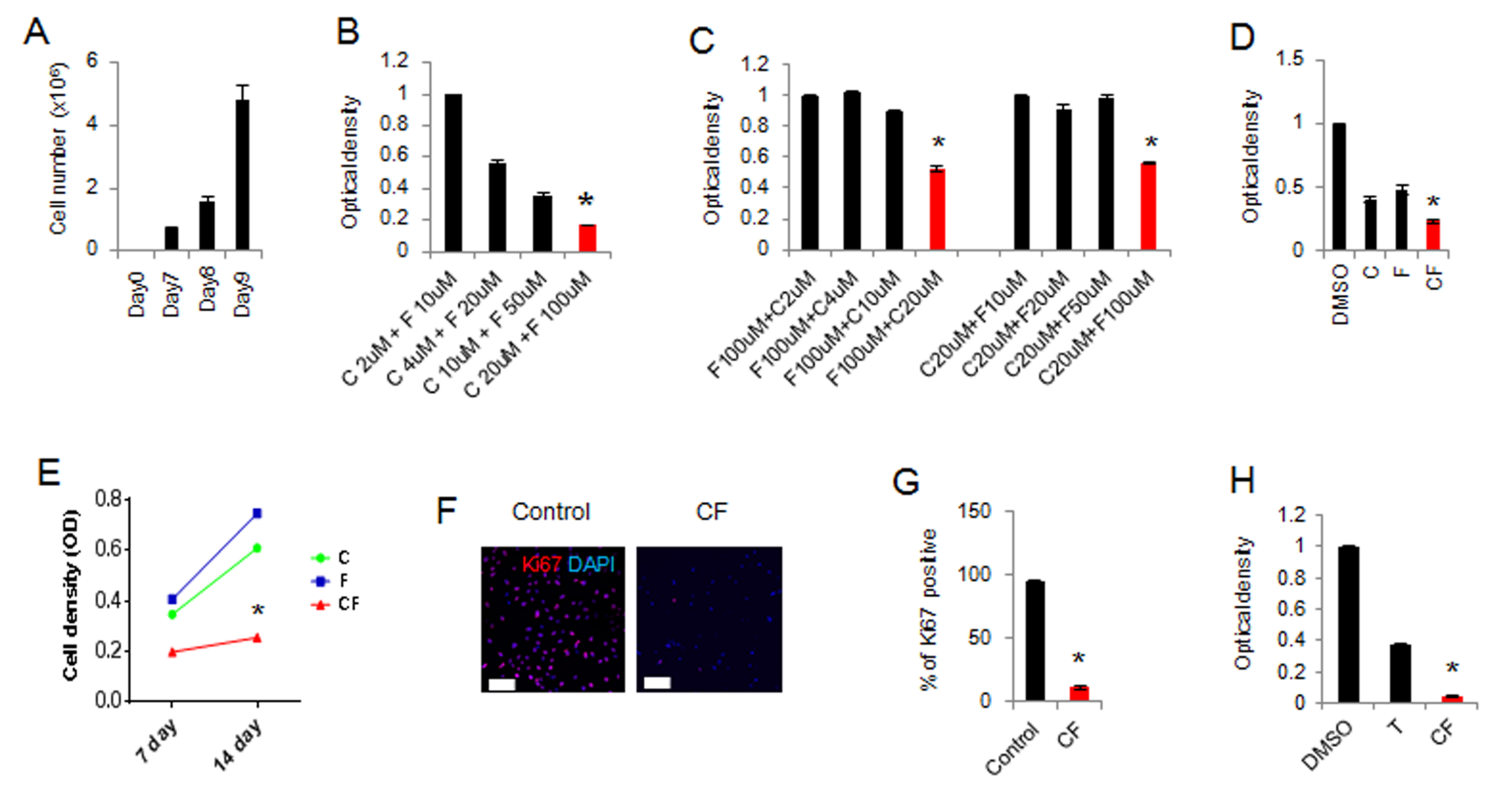
Growth suppression effect of a combination of CHIR99021 and forskolin in C6 glioma. (A) The proliferation rate of normal C6 glioma in neural induction media without small molecules. Cells were transferred to a new large dish at day 7. (B and C) Quantitative results of cell density at day 7 after neural induction. (D) Quantitative results of cell density at day 7 after neural induction. C, CHIR99021 (20 μM); F, forskolin (100 μM). (E) Quantitative results of cell density at days 7 and 14 days after neural induction with CHIR99021 (20 μM) and forskolin (100 μM). (F and G) Representative image and percentages of Ki67-positive cells in C6 glioma and SMiN cultures 35 days after neural induction with CHIR99021 (20 μM) and forskolin (100 μM). (H) Quantitative results of cell density at 7 days post-treatment. C, CHIR99021 (20 μM); F, forskolin (100 μM); T, temozolomide (50 μM). * indicates *p < 0*.*05*. Data are presented as the mean ± S.E.M. Scale bars represent 100 μm (F and G).

Next, we examined the ability of small-molecule treatments to suppress the proliferation of malignant glioma. The proliferation rate of C6 glioma cells is extremely fast, such that 100% confluence was achieved within 7 days, despite initial seeding at a low density (2 × 10^3^/cm^2^) (Fig 3A). We aimed to determine the concentrations at which maximum inhibition of cell proliferation could be achieved. We confirmed that a combination of 20 μM CHIR99021 and 100 μM forskolin significantly and powerfully inhibited C6 glioma cell proliferation (Fig 3B and 3C). Of note, the combination of 20 μM CHIR99021 and 100 μM forskolin produced a more powerful inhibition than any single-molecule treatment (Fig 3D and 3E). Next, we used Ki-67, a proliferating cell marker, to identify proliferating cells in glioma and SMiN cultures. While the most of control C6 glioma was Ki-67-positive, 11% Ki-67-positive cells were observed in SMiN cultures (Fig 3F and 3G). In addition, this inhibition was also greater than that of temozolomide (50 μM), a standard chemotherapeutic agent for treatment of brain tumors (Fig 3H). These results indicate that the combination of CHIR99021 and forskolin consistently and significantly suppressed the active proliferation of malignant glioma.

### Growth suppression effect of a combination of CHIR99021 and forskolin in patient-derived GBM

**Fig 4:**
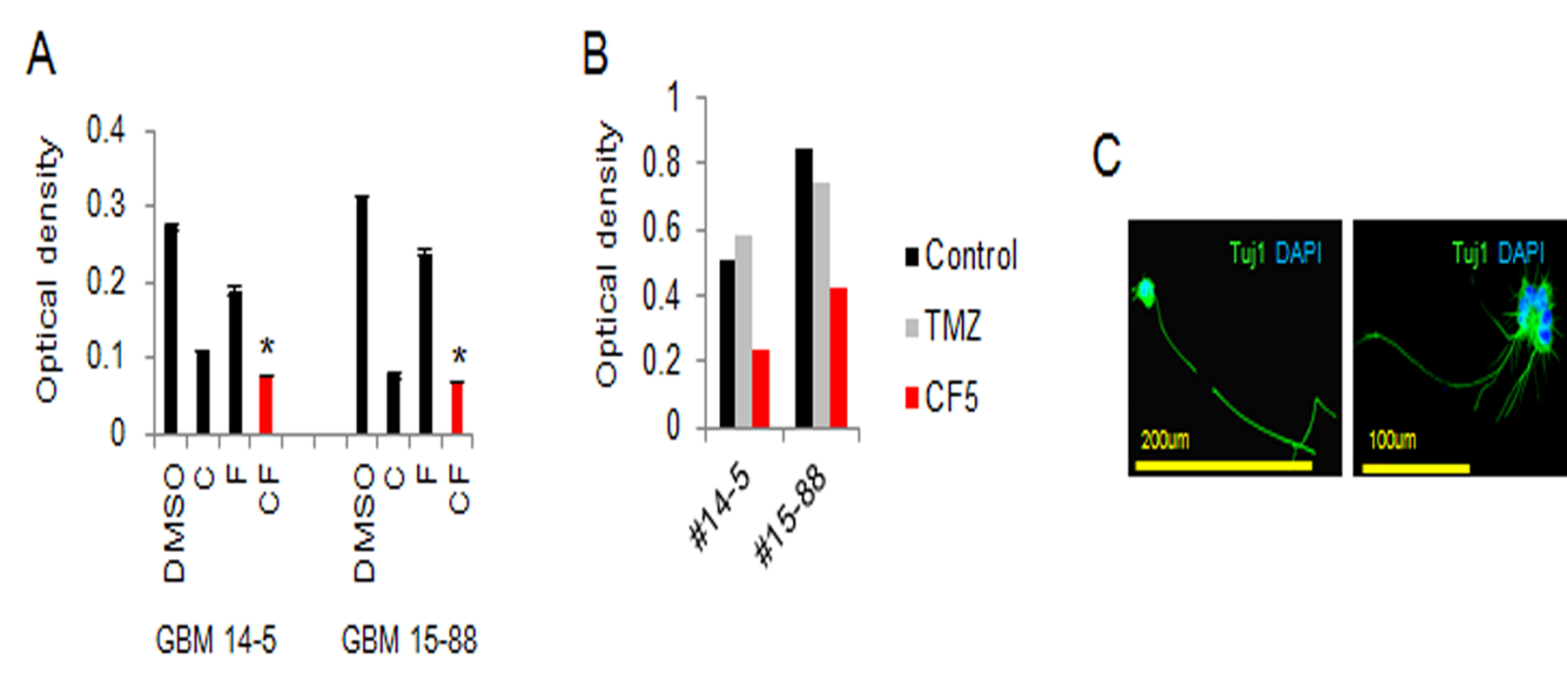
Growth suppression effect of a combination of CHIR99021 and forskolin in patient-derived GBM. (A) Quantitative results of cell density 7 days after neural induction. (B) Quantitative results of cell density 7 days post-treatment. C, CHIR99021 (10 μM); F, forskolin (50 μM); TMZ, temozolomide (50 μM). (C) Representative fluorescence image of Tuj1-positive cells 7 days after neural induction with CHIR99021 and forskolin. * indicates *p < 0*.*05*. Data are presented as the mean ± S.E.M. Scale bars represent 200 and 100 μm.

Next, we examined the ability of small-molecule treatments to suppress the proliferation of patient-derived GBM. We confirmed that a combination of 10 μM CHIR99021 and 50 μM forskolin powerfully inhibited GBM proliferation (Fig 4A), and the growth inhibition effect was also greater than that of temozolomide treatment (Fig 4B). The neuron-like morphology was also observed in GBM treated with CHIR99021 and forskolin (Fig 4C). These results indicate that the combination of CHIR99021 and forskolin induces growth inhibition in GBM.

### Gene expression profile of malignant glioma and SMiNs

**Fig 5:**
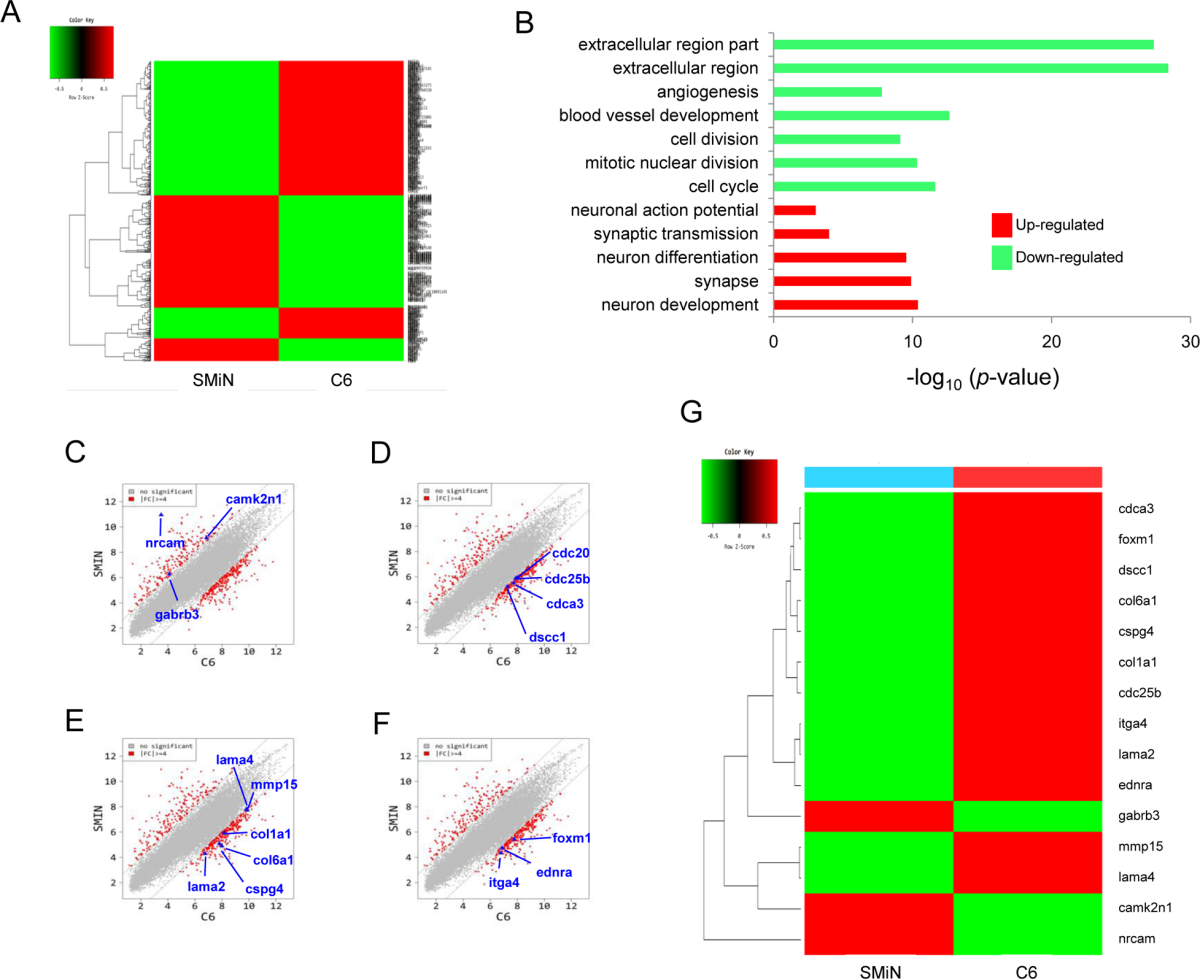
Gene expression profile of a malignant glioma and SMiN. (A) Heat map of significant gene in C6 glioma and SMiNs. (B) Up- or down-regulated gene ontology term in SMiNs. (C) Comparison of neuronal differentiation-related genes in C6 glioma and SMiNs. (D) Comparison of cell cycle-related genes in C6 glioma and SMiNs. (E) Comparison of ECM-related genes in C6 glioma and SMiNs. (F) Comparison of vessel development-related genes in C6 glioma and SMiNs. (G) Enriched gene list in C6 glioma and SMiNs.

We then investigated the gene expression profile regarding neural conversion and growth inhibition in C6 glioma and SMiNs. In a microarray analysis, we confirmed that gene ontology relating to cell differentiation, such as neuron development and projection, was up-regulated, and that neuronal genes were highly expressed in SMiNs (Fig 5B, 5C and 5G). In contrast, gene expression profiling and gene ontology relating to cell division and mitosis were markedly up-regulated in C6 glioma (Fig 5B, 5D and 5G). In addition, gene expression profiling and gene ontology relating to the extracellular matrix (ECM) and vessel development were significantly down-regulated in SMiNs (Fig 5B and 5E–5G). These results validated that CHIR99021 and forskolin induce both neural conversion and growth suppression on malignant glioma.

### Change of gene expression after treatment with CHIR99021 and forskolin

We analyzed the change of gene expression relating to cell cycle, glioma development, cytokine-cytokine receptor interaction, ECM-receptor interaction, cell adhesion molecule, PI3K-AKT, MAPK, and RAS signaling after treatment of small molecules (Table 1). Results indicate that the combination treatment with CHIR99021 and forskolin inhibits the cell cycle and glioma development, and lead to changes in cytokine-receptor interaction, ECM-receptor interaction and cell adhesion molecule. It indicates that changes in the PI3K/AKT and RAS/MAPK signaling pathways were induced by the neural conversion after treatment with CHIR99021 and forskolin.

**Table 1:** Change of gene expression relating to cell cycle, glioma development, cytokine-cytokine receptor interaction, ECM-receptor interaction, cell adhesion molecule, PI3K-AKT, MAPK and RAS signaling pathway after treatment of small molecules.

Related Pathway	Significantly down-regulated genes	Significantly up-regulated genes
Cell cycle	CCNA2 (-4.56), CDC25B (-4.03), PLK1 (-5.04), MCM7 (-4.10), CCNB2 (-5.06), PTTG1 (-5.35), CDC20 (-4.14), CDK4 (-2.94), CDK6 (-3.18)	NA
Glioma development	PDGFRA (-16.57), PDGFRB (-5.39)	NA
Cytokine-receptor interaction	MET (-4.29), PDGFRB (-5.39), PDGFRA (-16.57), CSF1 (-5.49)	NGFR (4.07)
ECM-receptor interaction	RELN (-7.27), ITGA1 (-4.96), SPP1 (-22.95), COL1A1 (-4.76), COL6A1 (-6.58), LAMA2 (-5.50), LAMA4 (-4.01), ITGA4 (-5.45), ITGB8 (-16.93)	NA
Cell adhesion molecule	SDC3 (13.1), CD28 (-5.10), RT1-S3 (-5.39), PTPRM (-4.25), ITGA4 (-5.45), ITGB8 (-16.93)	NRCAM (174.65)
PI3K/AKT signaling pathway	LPAR1 (-6.25), MET (-4.29), PDGFRB (-5.39), RELN (-7.27), ITGA1 (-4.96), PDGFRA (-16.57), SPP1 (-22.95), COL1A1 (-4.76), COL6A1 (-6.58), LAMA2 (-5.50), LAMA4 (-4.01), ITGA4 (-5.45), PCK2 (-4.37), ITGB8 (-16.93), CSF1 (-5.49), PRKAA2 (-4.06), NF-ҡB (-2.76)	NGFR (4.07)
MAPK signaling pathway	CDC25B (-4.03), PDGFRB (-5.39), NTRK2 (-7.48), PDGFRA (-16.57), RAPGEF2 (-4.26), RPS6KA5 (-4.57), MEF2C (-16.48)	NA
RAS signaling pathway	ETS1 (-5.26), MET (-4.29), PDGFRB (-5.39), PDGFRA (-16.57), CSF1 (-5.49), KRAS (-2.52), NRAS (-2.32)	NGFR (4.07)

### Anti-cancer effects of combined CHIR99021 and forskolin in a severe IMSCT model

**Fig 6:**
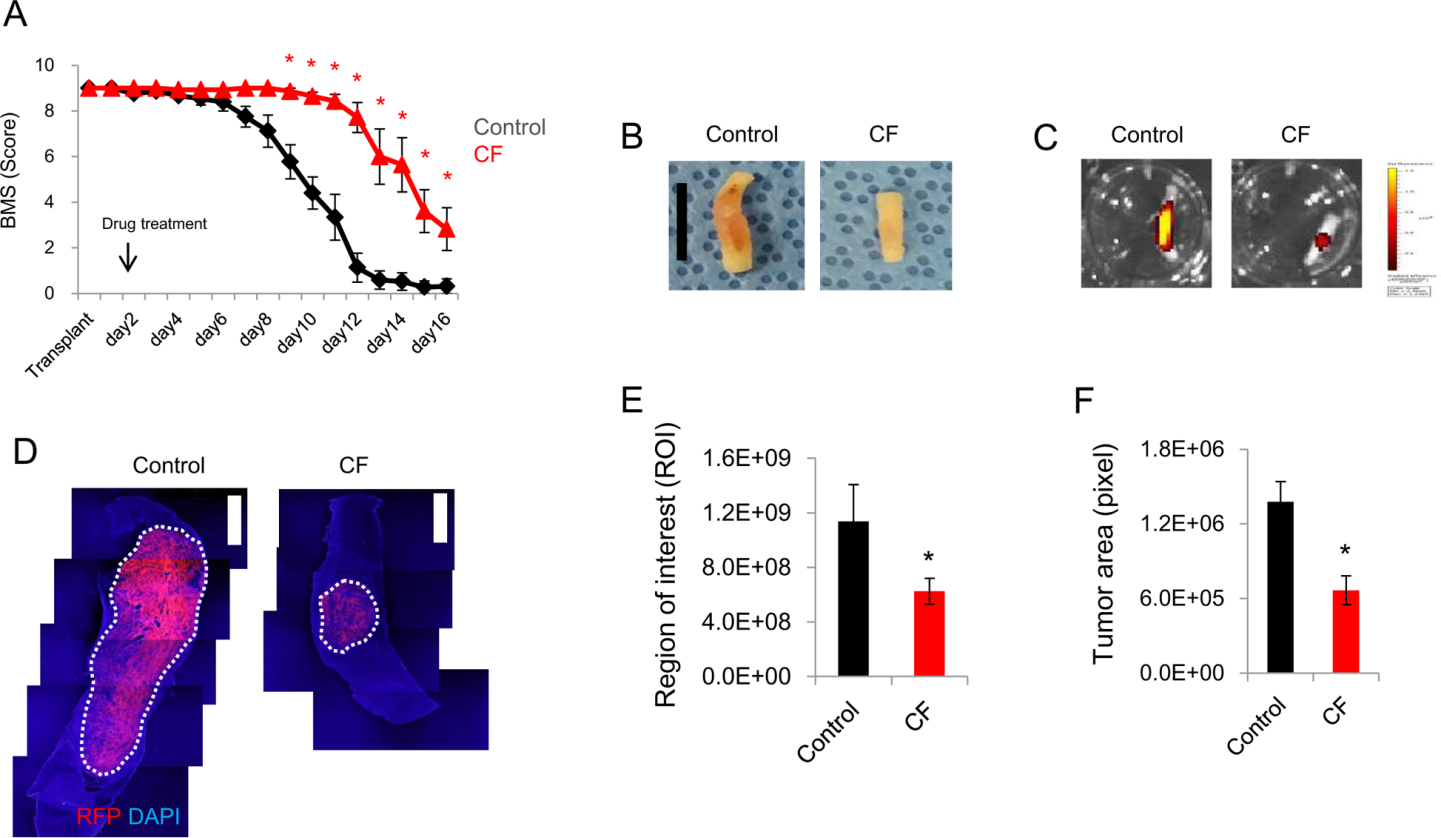
Anti-cancer effects of combined CHIR99021 and forskolin in a severe IMSCT model. (A) Neurologic function tests. (B) Representative image of dissected spinal cord at 16 days post-transplantation with RFP-expressing C6 glioma. (C) Representative optical imaging of dissected spinal cord at 16 days post-transplantation with RFP-expressing C6 glioma. (D) Representative image of sagittal sectioned-spinal cord at 16 days post-transplantation with RFP-expressing C6 glioma. (E) Quantitative result of ROI at 16 days post-transplantation with RFP-expressing C6 glioma. (F) Quantitative result of tumor area at 16 days post-transplantation with RFP-expressing C6 glioma. C, CHIR99021 (12.5 mg/kg); F, forskolin (10 mg/kg). * indicates *p* < 0.05. Asterisk means a significant difference to control. Data are presented as the mean ± S.E.M. Control (n = 11), CF (n = 7). Scale bars represent 1 cm (B) and 2 mm (D).

Next, we investigated whether the direct reprogramming technology based on the combination of small molecules can be applied to the treatment of malignant glioma. We verified the anti-cancer effect of the combination treatment with both CHIR99021 and forskolin on a severe IMSCT model. Interestingly, we confirmed that treatment with both CHIR99021 (12.5 mg/kg) and forskolin (10 mg/kg) markedly delayed the neurologic dysfunction in a severe IMSCT model (Fig 6A). By 16 days after glioma transplantation, the tumor volume had significantly decreased in the CHIR99021 and forskolin treated group (Fig 6B–6F).

## Discussion

It has been reported that fibroblasts can be directly reprogrammed into neurons through the forced expression of three pro-neuronal transcription factors [1]. This showed the possibility that a malignant tumor can be converted to post-mitotic cells through direct reprogramming. The direct conversion from a malignant tumor to benign cells is a potent therapeutic strategy for removing tumor cells because terminally differentiated cells will die from apoptosis. In line with this research, a previous study has shown that glioma can be converted to post-mitotic neurons via the forced expression of neural reprogramming factors such as Ngn2 and Sox11 [13]. However, intrinsic limitations related to chromosomal gene insertion persist. The present study is the first to show that glioma can be converted to neurons using small molecule reprogramming in the absence of neural transcription factors.

In a preliminary study, we investigated the neural conversion of glioma using a few small molecule combinations that have been previously used for neural induction, such as a83-01, dorsomorphin, CHIR99021, and forskolin. Seven days after combination treatment, CHIR99021 and forskolin showed higher neural conversion compared to other combinations such as A83-01/forskolin or A83-01/CHIR99021 and to any single treatment (Data not shown). In addition, we used neuronal differentiation media containing pro-differentiation reagents (such as N2, B27, BDNF, GDNF, and NT3) as a control. In the control group, we did not see any neural conversion. Thus, we concluded that the combined treatment with CHIR99021 and forskolin specifically induced neural conversion of glioma.

Neuronal direct reprogramming technology using a small molecule cocktail of 4–7 molecules was reported in fibroblasts [17,18]. Nevertheless, neither other authors nor we could confirm that fibroblasts can be converted into Tuj1-positive neurons by combination treatment with CHIR99021 and forskolin. In contrast, combination treatment with CHIR99021 and forskolin led to physiological and morphological changes in glioma. This result supports our hypothesis that the fate decision of glioma is more flexible than that of fibroblasts.

In a microarray analysis, we confirmed that gene ontology relating to cell differentiation was up-regulated in SMiNs. In contrast, gene expression profiling and gene ontology relating to cell division and mitosis was markedly up-regulated in C6 glioma. These results are the first to show the change of gene expression pattern relating to both neural conversion and growth suppression after neural conversion via small molecules. We did not observe the expression of any transcription factor implicated in neuronal conversion, such as Neurog2, after treatment with CHIR99021 and forskolin for 5 weeks. A previous study has shown that combination treatment with CHIR99021 and forskolin did not induce Neurog2 expression during neural conversion from fibroblast to neuron [17, 18]. It is possible that a mechanism other than the expression of transcription factors related to neural conversion is activated during C6 glioma neural conversion.

Further, it is unknown which signaling pathway was involved in glioma–neural conversion by forskolin and CHIR99021. In KEGG pathway analysis, we confirmed various changes in cell cycle, molecular interaction/information, and signaling pathways after small-molecule treatment.

In the cell cycle pathway, the *MCM* family, *CDK 4/6*, *PLK1*, and *CDC20* were markedly down-regulated in SMiNs. The expression level of *MCM*, a useful marker of cancer proliferation, is increased in different cancer types [21]. The depletion of *MCM7* inhibits glioblastoma proliferation [22]. It has been reported that the use of *CDK4/6* inhibitors can be applied for glioblastoma treatment [23]. In addition, the serine/threonine-protein kinase *PLK1*, a trigger of G2/M progression, is highly expressed in tumor cells [24]. These results indicate that the combination treatment with CHIR99021 and forskolin inhibits the cell cycle.

Small-molecule treatment led to changes of the pathways relating to glioma development and cytokine–receptor or ECM–receptor interaction. Especially, *PDGFR* was highly down-regulated in SMiNs. Thus, the downstream genes of *PDGFR* were also sequentially down-regulated. It is known that epidermal growth factor receptor (*EGFR*), *PDGFR*, and *Met* (hepatocyte growth factor receptor, *HGFR*) are expressed in glioma. Glioma expresses both the growth factor ligand and the receptor; hence, the autocrine and paracrine effects of growth factors may regulate proliferation in glioma. It has been reported that glioma growth, invasion, and angiogenesis signals are related to ECM components such as collagen, laminin, and integrin, and to receptor tyrosine kinase (RTK) genes such as *EGFR*, *PDGFR*, *IGF1R*, and *Met* [25]. Given the major role of ECM and RTK in glioma invasion and angiogenesis, down-regulation of genes relating to ECM-receptors or cytokine receptors interaction by CHIR99021 and forskolin may lead to the suppression of angiogenesis and glioma invasion *in vivo*.

Small-molecule treatment led to changes of RTK/PI3K/AKT and RAS/RAF/MAPK signaling pathways on glioma. The activated PDGF receptor triggers both the PI3K/AKT and RAS/RAF/ERK1/2 signaling pathways [25,26]. In the PI3K pathway, the PDGF expression increases the expression of *NF-ҡB* regulating cell proliferation and survival [25]. In addition, the PI3K/AKT pathway is implicated in glioma invasion [23]. It has been reported that transcription factors *c-jun*, *c-myc*, and *c-fos* are involved in cell proliferation. KEGG pathway analysis showed that these genes are involved in the MAPK pathway, and three transcription factors were down-regulated in SMiNs. These results indicate that the combination treatment with CHIR99021 and forskolin induced the neural conversion of glioma, and led to changes in the RTK/PI3K/AKT and RAS/RAF/MAPK signaling pathways involving cell growth and proliferation.

In a growth inhibition experiment in which small molecules were added to C6 glioma or GBM, GBM was more resistant to forskolin compared to C6 glioma. It is possible that some factors, such as cell type-specific differences or heterogeneity, may affect the results. Moreover, a previous study has shown that the drug response is different for each single clone of GBM [27]; Nevertheless, the combination of CHIR99021 and forskolin showed stronger growth inhibition effect compared to single treatment with either CHIR99021 or forskolin. However, the response to CHIR99021 and forskolin should be tested in various GBM clones. In the future, we plan to confirm our findings on GBM *in vivo*.

Collectively, combination treatment with small molecules led to various changes involving gene expression, cell-specific marker expression, signal transduction pathways, and physiological and morphological changes in glioma. These findings demonstrated that direct reprogramming technology based on small molecules can be applied to suppress the proliferation of malignant glioma. We suggest that a combination molecule comprising a GSK3 inhibitor and cAMP inducer would be a candidate molecule for the next-generation of anticancer drug, and that reprogramming technology is a potential therapeutic strategy that could potentially replace the therapeutic paradigm of the traditional treatment of malignant glioma.

## Supporting information

S1 Checklist Completed “The ARRIVE Guidelines Checklist” for reporting animal data in this manuscript. (DOCX)
